# European Academy of Nursing Science Summer Conference 2018: Leadership in Nursing: challenges for the future: Ghent, Belgium. 11-12 July 2018

**DOI:** 10.1186/s12912-018-0301-3

**Published:** 2018-08-17

## Keynote presentations

### K1 Leadership mentoring in nursing research, career development and scholarly productivity: what is already known?

#### Thóra B. Hafsteinsdóttir^1,2,3^ (t.hafsteinsdottir@umcutrecht.nl)

##### ^1^Associate Professor of Nursing, Department of Nursing Science, University of Utrecht, Utrecht, the Netherlands; ^2^Associate Professor of Nursing, Allied Health Care for people with chronic illnesses, University of Applied Sciences, Utrecht, The Netherlands; ^3^Visiting Professor of Nursing, Faculty of Nursing, University of Iceland, Reykjavik, Iceland

**Background**

Although nursing has been an academic discipline for decades, nursing research in many European countries was shown to be fragmented and postdoctoral nurses have difficulties with developing academic career. Lack of leadership is an important reason for this. The Dutch Leadership Mentoring in Nursing Research (LMNR) program was set up for postdoctoral nurses and their experiences with this program investigated.

**Aims**

To investigate leadership practices of postdoctoral nurses; explore post-doctoral nurses’ experiences with their leadership and career development; develop the LMNR program and to investigate the fellows’ expectations and experiences with the program, as well as the impact on their leadership practices.

**Methods**

A national survey investigated leadership practices of postdoctoral nurses, followed by a generic qualitative study exploring experiences of postdoctoral nurses working in academe; the LMNR-program developed, and qualitative study investigated the fellows experiences of the program.

**Results**

Dutch postdoctoral nurses have moderate leadership practices. Thirteen postdoctoral nurses were interviewed and five themes emerged: Taking the lead; Professional Identity; Supportive culture; Scientific World and Search for Balance. The LMNR program offers a two-year leadership and mentoring through which post-doctoral fellows extend competencies in leadership practices in research. Ten fellows finished the program who aspired furthering own research career, with a strong researcher and nurse identity. The one-year evaluations were positive, with participants describing changes in identity and leadership practice.

**Conclusions**

Leadership practices of postdoctoral nurses further need to be developed. Additional steps should be taken to further develop leadership and promote more academic career opportunities in order to strengthen nursing science. Leadership is highly important for successful career development and postdoctoral nurses use leadership to balance aspects in their career development. The structured and funded postdoctoral leadership program strengthened leadership competencies and career development and the fellows experienced the program as very successful. This is the first leadership and mentoring program in Europe and worldwide which focuses on postdoctoral nurses, and it represents an innovative way to strengthen nursing science.

### K2 Organisational development in coproduction between service users and staff: insights into achievements, challenges, and the contribution of nurses

#### Julie Repper^1,2,3^ (julie.repper@nottshc.nhs.uk)

##### ^1^Director of Implementing Recovery through Organisational Change (ImROC), Nottinghamshire Healthcare NHS Foundation Trust, Nottingham, UK; ^2^Recovery Lead, Nottinghamshire Healthcare NHS Foundation Trust, Nottingham, UK; ^3^Associate Professor of Recovery, University of Nottingham, Nottingham, UK

**Background**

Traditionally, nurses have been trained to ‘fix’ people; to have the answers to their questions: to be the experts. Increasingly, we are recognising that not only do we not have all the answers; but we are unable to fix everyone and we definitely are not the experts when it comes to enabling people to find the best ways of managing their own condition, their own treatment and most importantly, their own lives. As more people are living for longer and medical interventions become ever more effective, we are seeing many more people with long term, multiple and complex conditions, many of which are compounded by social factors such as poor housing, poor literacy, financial problems, relationship problems and mental health conditions. Whilst we might not be able to solve all of these problems, nurses have an important role in working in partnership with individuals, their families and their communities to facilitate greater self-management and control for the individual, greater confidence and support for family members, and more capable and inclusive communities.

In the presentation the unique position and role of nurses in creating co-productive partnerships that both value the contribution of all members and bring benefits for all involved are discussed. At an individual level coproduction is synonymous with shared decision making. At a team, service and community level, coproduction releases a huge additional set of resources and expertise that can enable people to recover and live well with meaningful roles and relationships within their communities.

**Methods**

The presentation will draw on evidence and practical examples from the ImROC programme (Implementing Recovery through Organisational Change) to illustrate ways in which nurses can lead coproduction to influence organisational values and purpose; achieve greater patient satisfaction, improve health outcomes and create more informed communities.

ImROC utilises a tried and tested methodology to achieve demonstrable change, including:

- Ten Organisational Challenges – to provide a baseline assessment, priority areas for change and develop coproduced action plans
- The PDSA (Plan Do Study Act) cycle in a continual process to guide and review change at every level of the organisation
- Recovery focused quality improvement interventions (such as the Team Recovery Implementation Plan; the employment of peer workers; the development of Recovery Colleges; the introduction of coproduction structures) to inform and evaluate change
- Recovery focused/transformational leadership approaches
- The facilitation of local action learning sets
- The production of accessible, evidence based, coproduced and free to download Briefing papers to inform best practice.

**Conclusions**

Nurses have a key role in enabling these changes. Owing to their training, experience, values and the size of the nursing workforce within health and social care organisations they are pivotal in the success of initiatives to develop Recovery focused cultures and practices. However, these developments are transformational and require a shift away from traditional practices so nurses will need coaching, training and mentoring to support them to lead and sustain these improvements.

### K3 Advanced practice nursing roles in Europe

#### Claudia B Maier^1,2,3^ (c.maier@tu-berlin.de)

##### ^1^Postgraduate researcher, Department of Healthcare Management, Technische Universität Berlin, Berlin, Germany; ^2^Senior fellow, Center for Health Outcomes and Policies, University of Pennsylvania, Philadelphia, PA, USA; ^3^Consultant, World Health Organisation (WHO), Geneva, Switzerland

**Background**

Health and nursing care has become increasingly complex, due to technological advances, more complex treatment and multimorbidity on the rise. The study aimed to analyse the roles of Advanced Practice Nurses (APN) in Europe, related reforms and implementation.

**Methods**

International study, based on the TaskShift2Nurses expert survey in 39 countries, including 93 experts, a literature review, and routine data on Nurse Practitioners (NP).

**Results**

Eleven countries had extensively expanded the scopes-of-practice for NP/APN. The size of the NP workforce is small, but increasing rapidly, at a higher rate than among the physician workforce; this is partly linked to the rise in educational programmes to train nurses in advanced roles. Many countries in Europe (e.g. Cyprus, Estonia, Finland, France, Netherlands, Poland, Spain) have adopted new laws since 2010 to authorise nurses to prescribe medicines, but the number of medicines and levels of independence vary considerably.

**Conclusions**

Many countries are expanding the roles for nurses, some with major reforms underway, others slowly progressing. Overcoming policy barriers is key to ensure a smooth transition for nurses, teams and patients.

**References**

1. Maier CB, Aiken LH, Busse R (2017). *Nurses in advanced roles. Policy Levers for Implementation.* OECD Working Paper.November 2017. http://www.oecd-ilibrary.org/social-issues-migration-health/nurses-in-advanced-roles-in-primary-care_a8756593-en

## Speaker presentations

### S1 EFFIP (E-support for Families & Friends of Individuals affected by Psychosis) – developing and evaluating an eHealth complex intervention for family carers

#### Jacqueline Sin^1^, Claire Henderson^2^, Aurora Sesé^3^, Luke Woodham^3^, Kavirthana Krishnamoorthy^3^, Steve Gillard^1^

##### ^1^Population Health Research Institute, St George's, University of London, London, UK; ^2^Institute of Psychiatry, Psychology & Neuroscience, King's College London, London, UK; ^3^e-Learning Unit, Institute of Medical and Biomedical Education, St George's, University of London, London, UK

###### **Correspondence:** Jacqueline Sin (jasin@sgul.ac.uk)

**Background**

Psychosis including schizophrenia are the most common severe mental illness. [1,2] Coping with psychosis is often challenging and difficult not just for the individuals themselves but everyone close to them. [3,4,5] The EFFIP project is a five-year research programme aiming to develop and evaluate an online intervention providing support, information, and coping strategies to promote the wellbeing of carers of people affected by psychosis.

**Methods**

This nurse-led project enlists a team of multi-disciplinary clinical and academic experts, digital health professionals, people with lived experience of psychosis, carers and key stakeholders. We use a mixed methods study design based on the UK Medical Research Council’s framework for the development, modelling and piloting, and evaluation of complex interventions.[6]

**Results and conclusions**

Over the last two years, we have completed two systematic reviews [4,5] and focus group studies with carers and service users, to map out the design and content of the intervention. We built the intervention prototype and ran a usability study to test its usability and to further refine it through an iterative consultation process with carers and extensive Public and Patient Involvement (PPI) activities. Third and lastly, we just launched a randomised controlled trial to evaluate the effectiveness of the online intervention in promoting carers’ wellbeing and caregiving experiences (ISRCTN 89563420).

**Acknowledgements**

We also acknowledge all the inputs and contributions from the Project Reference Group and Expert Advisory Group members – see http://cope-support.org/team/

**References**

1. NICE (2014) *Psychosis and schizophrenia in adults: Treatment and management* (CG 178). London: The British Psychological Society & The Royal College of Psychiatrists.
2. The Schizophrenia Commission (2012) *The Abandoned Illness: A Report from the Schizophrenia Commission.* London: Rethink Mental Illness.
3. Pharoah F., Mari J., Rathbone J. & Wong W. (2010) Family intervention for schizophrenia. Cochrane *Database of Systematic Reviews Issue* 12. Art. No.:CD000088. DOI:10.1002/14651858. CD000088.pub3.
4. Sin J., Gillard S., Spain D., Cornelius V., Chen T. & Henderson C. (2017) Effectiveness of psychoeducational interventions for family carers of people with psychosis: A systematic review and meta-analysis. *Clinical Psychology Review*. 56: 13-24.
5. Sin J., Henderson C., Spain D., Cornelius V., Chen T. & Gillard S. (2018) eHealth interventions for family carers of people with long term illness: A promising approach? *Clinical Psychology Review*. https://doi.org/10.1016/j.cpr.2018.01.008.
6. Medical Research Council (2008) *Developing and Evaluating Complex Interventions: New Guidance.* Retrieved from: http://www.mrc.ac.uk/documents/pdf/complex-interventions- guidance/(last accessed on 27th July 2017).

### S2 Advancing the science of literature reviewing in nursing: the Focused Mapping Review and Synthesis as a novel approach

#### Caroline Bradbury-Jones, Julie Taylor

##### School of Nursing, University of Birmingham, Birmingham, UK

###### **Correspondence:** Caroline Bradbury-Jones (c.bradbury-jones@bham.ac.uk)

**Background**

Literature reviews are an important and popular part of synthesising evidence in nursing [1]. There are numerous approaches, each with their distinctive features and purposes [2]. Most types of review involve exhaustive searching and retrieval of as much relevant evidence as possible on a particular subject and from this, firm conclusions can be drawn or gaps in evidence identified. We present and critique a new form of review: The ‘Focused Mapping Review and Synthesis’ (FMRS).

**Methods**

Using illustrative examples from our published FMRS papers and extant literature on leadership, we present this new review type. In line with the conference theme, we use leadership in nursing as an example.

**Results**

Rather than synthesising evidence of ‘what works’, a FMRS approach aims to identify the contours, boundaries or assumptions associated with a subject within a specific body of literature.

**Conclusions**

FMRS offers a useful addition to the methodological toolkit of researchers.

**References**

1. Aveyard H (2010) Doing a Literature Review in Health and Social Care: A Practical Guide. Berkshire: Open University Press.

2. Grant MJ, Booth A (2009) A typology of reviews: an analysis of 14 review types and associated methodologies. Health Information & Libraries Journal, 2009, 26(91-108).

### S3 Development of a psycho-educational intervention program in oncology according to the complex interventions methodology

#### Otília Barreto^1,2^, Adriana Henriques^2^

##### ^1^Regional Health Service of the Autonomous Region of Madeira, Madeira, Portugal; ^2^University of Lisbon and School of Nursing of Lisbon, Lisbon, Portugal

###### **Correspondence:** Otília Barreto (maria.barreto@campus.esel.pt)

**Background**

The diagnosis of cancer disease has a great negative impact on both the patient and his/her family. This disease implies the adoption of coping strategies in order to cope with it. Some people need the specialized intervention of health professionals to adopt effective strategies to deal with the disease.

**Methods**

The development of the psychoeducational intervention program followed the guidelines of the Medical Research Council. This study covered the Development Phase and the Feasibility Phase of the Complex Interventions Model. In each phase the mixed method was used. We did two Systematic Reviews of Literature, submitted the program to expert peers and a Focus Group at the end of the program with the participants.

**Results**

The final result of this process of construction of the psychoeducational program gave rise to a multifamily group intervention program. The program's contents cover the concept of cancer, the demystification of this disease, adaptation to cancer, strategies to deal with anxiety and stress, family adaptation to cancer and lastly the resources of support that can appeal throughout the process of disease.

**Conclusions**

This construction process allows us to conclude this specific intervention as a working tool for nurses who develop their professional activity, and to change the current nursing care.

### S4 Nurses’ working context as an antecedent of autonomy-supportive communication - the contribution of the Self-Determination Theory

#### Veerle Duprez^1^, Maarten Vansteenkiste^2^, Dimitri Beeckman^1^, Sofie Verhaeghe^1^, Ann Van Hecke^1^

##### ^1^University Centre for Nursing and Midwifery, Department of Public Health, Faculty of Medicine and Health Sciences, Ghent University, Ghent, Belgium; ^2^Developmental Psychology, Department of developmental, personality and social psychology; Ghent University, Ghent, Belgium

###### **Correspondence:** Veerle Duprez (Veerle.Duprez@UGent.be)

**Background**

Within chronic care, it is generally accepted that patients are empowered to take the lead in their chronic condition management. A clincal leadership needs to be established, characterized by a collaborative partnership between the patient and the nurse. However, nurses sometimes leave little room for an approach that aknowledges a central role to patient participation and autonomy.

**Methods**

Rooted in the Self-Determination Theory, this study addressed (1) nurses’ autonomy-supportive approach, (2) whether work-related antecedents predict their approach while supporting patients towards self-management, and (3) the possible mediating role of burnout in these effects by structural equation models. A multicentre study was conducted within chronic care nurses (N=509).

**Results**

A two-dimensionality differing in person-centeredness and directiveness was present, i.e. autonomy-supportive, structuring, controlling, or chaotic approach. Antecedents indicated that nurses who experienced autonomy, competence and relatedness in their work context tended more to an autonomy-supportive approach. Nurses’ motives for self-management support differently predict the use of autonomy-supportive or more controlling approaches.

**Conclusions**

Managers face the challenge to provide job autonomy, as well as endorse the value of self-management support, which in turn leads to nurses’ provision of autonomy to patients.

### S5 Coproduction of knowledge for practice through a participatory action research and process evaluation project (Lydia Osteoporosis Project 2, LOP 2)

#### Margaret Coulter Smith^1^, Anthony Schrag^2^, Fiona Kelly^1^, Claire Pearson^1^, Alison Bacigalupo^1^

##### ^1^Division of Nursing, School of Health Sciences, Queen Margaret University, Edinburgh, Scotland, UK; ^2^Division of Media, Communication and Performing Arts, School of Arts, Social Sciences and Management, Queen Margaret University, Edinburgh, Scotland, UK

###### **Correspondence:** Margaret Coulter Smith (msmith1@qmu.ac.uk)

**Background**

Participatory action research (PAR) is active, collaborative and seeks to develop knowledge from everyday occurrences (Reason and Bradbury 2013). A creative movement workshop developed from the Lydia Osteoporosis PAR Project 2 (LOP 2) and enabled volunteer local research collaborators and participants to articulate new practice knowledge.

**Methods**

A workshop co-designed by nurse researchers and artists as facilitators aimed to explore beliefs, understandings, mental models, and ‘tacit’ knowledge about osteoporosis and fracture risk in an ageing population (IOF 2017, SIGN 2015). Theoretical frameworks included ‘Embodied cognition’ (Balcetis and Cole 2009), ‘model-based learning environments’ (Pirnay-Dummer et al, 2012), biomedical knowledge, and creative arts.

**Results**

Workshop participants (N=19) progressed through activities ranging from individual drawings of human figures to more physical movement tasks influenced by ‘embodied cognition’, with tissue paper ‘exoskeletons’ as a metaphor for fracture risk. The activities created dissonance and puzzlement to challenge beliefs and knowledge structures. Multiple materials were used. Processes and creative outputs were documented in photographs and text. Data analysis was undertaken and a metaphor appeared to convey practice when assisting with mobility ‘Mobilising with osteoporosis is like a dance. Sometimes you will lead. Sometimes you will follow’.

**Conclusions**

Workshop was positively evaluated.

### S6 From qualitative meta-summary to qualitative meta-synthesis: developing a new situation-specific theory of barriers and facilitators for self-care in heart failure patients

#### Oliver Rudolf Herber (Oliver.Herber@med.uni-duesseldorf.de)

##### Institute of General Practice (ifam), Medical Faculty of the Heinrich Heine University Dusseldorf, Dusseldorf, Germany

**Background**

Situation-specific theories provide nurses with a vehicle to guide their decisions or make assumptions. Yet, a theory on barriers and facilitators for self-care in heart failure patients is absent in spite of sufficient primary qualitative research required to execute a qualitative meta-synthesis represented as a situation-specific theory. The purpose is to describe the development of a situation-specific theory of barriers and facilitators to heart failure self-care.

**Methods**

We employed meta-synthesis techniques to integrate statements of findings pertaining to barriers and facilitators to heart failure self-care that were derived previously through meta-summary techniques.

**Results**

According to the proposed theoretical model, self-care behaviour is the result of a patient’s decision-making process. This process is mainly influenced by two key concepts: ‘self-efficacy’ and the ‘patient’s disease concept.’ Facilitative and inhibitive factors have been identified influencing these two key concepts as well as the decision-making process itself, thereby either enabling or hampering the execution of effective heart failure self-care.

**Conclusions**

A situation-specific theory of barriers and facilitators to heart failure self-care provides a blueprint for nursing practice. Further research is needed to validate the model through empirical testing. Once fully matured, the model may be useful in developing behavioural interventions.

### S7 Leadership competencies and attributes in advanced nursing practice: an integrated review

#### Maud Heinen^1^, Anita Huis^1^, Hester Vermeulen^2^, Catharina van Oostveen^2^, Jeroen Peters^2^

##### ^1^IQ healthcare, Radboud Institute for Health Sciences, Radboud University Medical Centre, Nijmegen, the Netherlands; ^2^University of Applied Sciences, Nijmegen, the Netherlands

###### **Correspondence:** Maud Heinen (m.machiels@maastrichtuniversity.nl)

**Background**

Developments in healthcare and within the nursing profession ask for well trained nurses with leadership qualities at all levels of the healthcare system. Clinical nurses who are trained at master’s level, e.g. Advanced Practice Nurses (APNs) and Clinical Nurse Leaders (CNLs) are ideally positioned to lead health care reform in nursing. Identifying leadership competencies and attributes internationally is the first step for an evidence based curriculum on leadership.

The aim of this study is to contribute to defining leadership by establishing what leadership competencies and attributes are expected of the Master level educated nurse (APN & CNL) from an international perspective.

**Methods**

An integrative review design was used which allows for the combination of various methods to synthesize the findings of included studies and literature. The Embase, Medline and Cinahl databases and websites of international professional nursing organizations were searched for publications on leadership competencies.

**Results**

Seventy-eight competencies were identified in ten studies and six frameworks. Synthesis of results led to the identification of 26 core competencies within the clinical, professional, health systems and health policy leadership domains (Hamric et al 2014). The least number of competencies were identified in the health policy domain.

**Conclusions**

This model of leadership core competencies could be used as a solid base for international leadership development.

### S8 Clinical leadership: how can we define and recognize leaders in bedside nursing care?

#### Nele De Roo, Sabrina Nachtergaele

##### Artevelde University College Ghent, Nursing Department, Ghent, Belgium

###### **Correspondence:** Nele De Roo (nele.deroo@arteveldehs.be)

**Background**

Nurses have a considerable role in coordinating and implementing interdisciplinary care in hospitals in order to guarantee quality of care (QoC). Therefore every nurse has to possess leadership qualities, including nurses without a formal leadership role.

The aim of this study was to define the concept of clinical leadership and to understand how clinical leaders can be recognized within a nursing team.

**Methods**

A literature research was conducted combined with in-depth interviews with experts and semi-structured focus groups. A qualitative content analysis of the transcribed expert and focus group interviews was done.

**Results**

The concept ‘clinical leadership’ involves bedside nurses who deliver daily care, act as role models and influence, motivate and inspire others with their values and beliefs to improve patient care, without having formal authority. Characteristics like creativity, clinical expertise, flexibility, communication skills and vision towards the future are linked to this concept. There is a positive correlation between the presence of these clinical leaders and QoC. Senior nurses acknowledge and recognize these leadership qualities in bedside nurses, but nurses do not recognize this within themselves.

**Conclusions**

Understanding of this concept is necessary to raise leadership-self-awareness in bedside nurses, which has a positive impact on QoC.

### S9 Nursing in the governance model of health programmes

#### Andreia Silva da Costa^1^, Adriana Henriques^2^

##### ^1^Directorate of Disease Prevention and Health Promotion, Directorate-General of Health, Lisbon, Portugal; ^2^ESEL – Escola Superior de Enfermagem de Lisboa e ISAM – Instituto Superior Ambiental da Faculdade de Medicina Preventiva de Lisboa, Lisbon, Portugal

###### **Correspondence:** Andreia Silva da Costa (andreiasilva@dgs.min-saude.pt)

**Background**

The epidemiological and demographic patterns of transition present shifting challenges to European health systems. Aging and higher prevalence of non-communicable diseases challenge health services organisations.

Attribution of responsibilities and activities amongst professionals and their interactions can be adapted, creating new strategies and governance models implying nurses can equate national programmes from management to execution.

This paper aims to describe good practices in Portugal reflecting national programmes implemented locally by nurses articulated with multiple stakeholders.

**Methods**

The methodology used involved detailed analysis of governance models available on health ministry websites and monitoring reports showing process and outcome indicators of national, regional and local programmes managed by nurses.

**Results**

Analysis revealed programmes nationally coordinated by nurses, namely the National Programme for Children and Youth Health. This Programme provides health surveillance from birth through interventions of health professionals. Indicators related to health promotion and disease prevention revealed positive results and its indicators place Portugal positively when compared to European countries.

**Conclusions**

The dissemination of these governance models is considered relevant for the recognition of nurses in the coordination of programmes at local and national levels, specifically related to child health and vaccination, as health gains obtained place Portugal amongst European countries with the best results in this field.

### S10 Core Outcome Set (COS) for health- and nursing research: the case of incontinence-associated dermatitis (IAD)

#### Karen Van den Bussche, Dorien De Meyer, Dimitri Beeckman

##### ^1^Skin Integrity Research Group, University Centre for Nursing and Midwifery, Department of Public Health, Ghent University, Ghent, Belgium

###### **Correspondence:** Karen Van den Bussche (karen.vandenbussche@ugent.be)

**Background**

In health- and nursing science, clinical trials contribute to evidence-based practice and informed clinical decision making. However, trial results are often contradictory due to varying methodological quality, populations, interventions, and outcomes. The lack of comparability of clinical trial outcomes is a major challenge in the field of IAD prevention and treatment. Numerous initiatives to standardise outcomes have been launched since the 1990s. Today, the concept of a Core Outcome Set (COS) is widely accepted.

**Methods**

A list of outcome domains was generated through a systematic literature review, consultation of experts and three patient interviews. The project team reviewed and refined the outcome domains prior to starting a three-round Delphi procedure. The panellists, including healthcare providers, researchers and industry were invited to rate the importance of the outcome domains.

**Results**

We extracted 1852 outcomes from 244 articles. Experts proposed 56 and patients 32 outcomes. After refinement, 57 panellists from 17 countries rated a list of 58 outcome domains. The final list of outcome domains includes erythema, erosion, maceration, IAD-related pain, and patient satisfaction.

**Conclusions**

The use of a COS in clinical trials will contribute to international standardisation, comparability between trials, patient-centred care, and informed clinical decision making.

### S11 Experiences with a nurse-led self-management support intervention for people with chronic conditions; a mixed-methods approach

#### AnneLoes van Staa^1^, Janet Been-Dahmen^1^, Heleen van der Stege^1^, Denise Beck^2^, Mirjam Tielen^2^, Marleen van Buren^2^, Cora Braat^3^, Emma Massey^2^, Wendy Oldenmenger^3^, Erwin Ista^2^

##### ^1^Department of Healthcare & Welfare, NHL University of Applied Sciences, Leeuwarden, the Netherlands; ^2^Erasmus University Medical Centre, Dept. of Internal Medicine, Rotterdam, the Netherlands; ^3^Erasmus Medical Centre Cancer Institute, Rotterdam, the Netherlands

###### **Correspondence:** AnneLoes van Staa (a.van.staa@hr.nl)

**Background**

Self-management support is a core task for nurses, but it is unclear what it entails. Using Intervention Mapping, a nurse-led intervention was developed: people with kidney transplant (KT) and head-neck cancer (HNC) indicated their support needs. The intervention combines the assessment of self-management challenges using a conversational tool (Self-Management Web) with solution-focused communication techniques, including goal-setting, action-planning and monitoring progress. This pilot-study aimed to evaluate the feasibility and first effects of the intervention.

**Methods**

A controlled before-after evaluation study was conducted in 2 outpatient clinics of a Dutch University Hospital. To gain insight into fidelity, feasibility, and acceptability, we did interviews and observations. Quantitative methods consisted of pre-post surveys within-group comparison; and intervention (n=23 KT; n=27 HNC) versus historic control (n=43 KT; n=28 HNC) between-group comparison.

**Results**

The intervention was highly valued because of the open, holistic focus. It helped patients to build a relationship of trust with the nurse and made them more competent in problem-solving skills. Quantitative analyses indicated a significant change in self-efficacy (HNC) and in patient-centredness of nursing care (KT) but not in self-management skills.

**Conclusions**

This holistic self-management support intervention seems a promising tool to help chronic patients deal with daily life challenges.

### S12 Leadership activities and associated competencies of advanced practice nurses and midwives: a cross-sectional study

#### Regine Goemaes^1^, Elsie Decoene^2^, Dimitri Beeckman^1^, Sofie Verhaeghe^1,3^, Ann Van Hecke^1,4^

##### ^1^Department of Public Health, Faculty of Medicine and Health Sciences, Ghent University, Ghent, Belgium; ^2^Cancer Centre, Ghent University Hospital, Ghent, Belgium; ^3^Vives University College, Roeselare, Belgium; ^4^Nursing Science, Ghent University Hospital, Ghent, Belgium

###### **Correspondence:** Regine Goemaes (Regine.Goemaes@UGent.be)

**Background**

Advanced practice nursing and midwifery roles are increasingly implemented internationally. Although leadership is an important part of these roles, research regarding leadership activities of advanced practice nurses and midwives within and beyond healthcare organizations is scarce.

**Methods**

A questionnaire-based, cross-sectional study was undertaken among advanced practice nurses and midwives in hospitals to examine leadership activities and associated, self-reported competencies. Activity and competency level frequencies were calculated. Regression analysis identified factors associated with task non-execution.

**Results**

Advanced practice nurses and midwives (n=63) mainly focus on guideline and care protocol development within the hospital, maintaining contacts with colleagues in other healthcare organizations and participating in policy development meetings regarding domain-specific topics. A minority of advanced practitioners participate in hospital (department) policy meetings and (inter)national advisory boards, or maintain contacts with international professional associations and patient organizations. Non-execution of several leadership activities was associated with advanced practitioners feeling incompetent.

**Conclusions**

As feeling incompetent was associated with task non-execution, optimization of advanced practice nurses and midwives’ training regarding leadership activities should be considered. Leadership competencies are essential for the advancement of nursing and midwifery on a national and international level and for the further professionalization of nursing and midwifery policy.

### S13 Job satisfaction in relation to communication among nurses

#### Peter Vermeir^1,2^, Sophie Degroote^1^, Dominique Vandijck^1,3,4^, Rik Verhaeghe^2,3^, Dirk Vogelaers^1,2^, An Mariman^1,2^

##### ^1^Department of General Internal Medicine, Ghent University Hospital, Ghent, Belgium; ^2^Faculty of Medicine and Health Sciences, Ghent University, Ghent, Belgium; ^3^Department of Public Health, Ghent University, Ghent, Belgium; ^4^Department of Business Economics, Hasselt University, Diepenbeek, Belgium

###### **Correspondence:** Peter Vermeir (peter.vermeir@uzgent.be)

**Background**

Within the context of a worldwide nurse shortage and high turnover rates, improving nursing effectiveness is an important goal.

**Methods**

Literature review.

**Results**

Inter-professional teamwork is achieved by the interactive efforts of all team members involved through good communication and respect for the role of the other team members. There should be room for the contribution of each team member.

Communication impacts job satisfaction and job satisfaction impacts nurses’ turnover, which negatively influences quality of care. Furthermore, patient satisfaction is also influenced by nurses’ job satisfaction.

Inter-professional education which focuses on helping teams to communicate in appropriate and effective ways, is needed. Examples are: TeamSTEPPS (inter-professional communication training model), learning from errors and the SBAR structure.

**Conclusions**

Communication- and job satisfaction are the result of a complex and multifactorial interaction, involving both internal and external factors. Job dissatisfaction is a reliable predictor for turnover. Achieving a balance is important for enabling job satisfaction which is required for both organizational stability and for guaranteeing patient safety. This can only be achieved through an organization wide multimodal prevention and intervention program which aims to optimize different modalities of inter-professional communication, workload and job satisfaction.

