# Correction to: S11 Experiences with a nurse-led self-management support intervention for people with chronic conditions; a mixed-methods approach

**DOI:** 10.1186/s12912-019-0327-1

**Published:** 2019-02-03

**Authors:** AnneLoes van Staa, Janet Been-Dahmen, Heleen van der Stege, Denise Beck, Mirjam Tielen, Marleen van Buren, Cora Braat, Emma Massey, Wendy Oldenmenger, Erwin Ista

**Affiliations:** 1 Rotterdam University of Applied Sciences, Research Centre Innovations in Care, Rotterdam, The Netherlands; 2 Deparment of Internal Medicine, Erasmus University Medical Centre, Rotterdam, the Netherlands; 3 Erasmus Medical Centre Cancer Institute, Rotterdam, the Netherlands

**Correction to: BMC Nurs 2018, 17(Suppl 1)**

**https://doi.org/10.1186/s12912-018-0301-3**

After publication of this supplement [1], it was brought to our attention that in abstract S11, the author affiliation No. 1 is incorrect. It should be ‘Rotterdam University of Applied Sciences, Research Centre Innovations in Care, Rotterdam, The Netherlands’, but not ‘Department of Healthcare & Welfare, NHL University of Applied Sciences, Leeuwarden, the Netherlands’.

## S11 Experiences with a nurse-led self-management support intervention for people with chronic conditions; a mixed-methods approach

AnneLoes van Staa^1^, Janet Been-Dahmen^1^, Heleen van der Stege^1^, Denise Beck^2^, Mirjam Tielen^2^, Marleen van Buren^2^, Cora Braat^3^, Emma Massey^2^, Wendy Oldenmenger^3^, Erwin Ista^2^

1. Department of Healthcare & Welfare, NHL University of Applied Sciences, Leeuwarden, the Netherlands;
2. Erasmus University MedicalCentre, Dept. of Internal Medicine, Rotterdam, the Netherlands;
3. ErasmusMedical Centre Cancer Institute, Rotterdam, the Netherlands

Correspondence: AnneLoes van Staa (a.van.staa@hr.nl)

BMC Nursing 2018, 17(Suppl 1):S11

## Background

Self-management support is a core task for nurses, but it is unclear what it entails. Using Intervention Mapping, a nurse-led intervention was developed: people with kidney transplant (KT) and head-neck cancer (HNC) indicated their support needs. The intervention combines the assessment of self-management challenges using a conversational tool (Self-Management Web) with solution-focused communication techniques, including goalsetting, action-planning and monitoring progress. This pilot-study aimed to evaluate the feasibility and first effects of the intervention.

## Methods

A controlled before-after evaluation study was conducted in 2 outpatient clinics of a Dutch University Hospital. To gain insight into fidelity, feasibility, and acceptability, we did interviews and observations. Quantitative methods consisted of pre-post surveys within-group comparison; and intervention (*n* = 23 KT; *n* = 27 HNC) versus historic control (*n* = 43 KT; *n* = 28 HNC) between-group comparison.

## Results

The intervention was highly valued because of the open, holistic focus. It helped patients to build a relationship of trust with the nurse and made them more competent in problem-solving skills. Quantitative analyses indicated a significant change in self-efficacy (HNC) and in patient-centredness of nursing care (KT) but not in self-management skills.

## Conclusions

This holistic self-management support intervention seems a promising tool to help chronic patients deal with daily life challenges.
